# Colonization with antibiotic resistant bacteria in communities and hospitals across six countries, including Bangladesh, Botswana, Chile, Guatemala, India, and Kenya

**DOI:** 10.1038/s41598-025-94750-3

**Published:** 2025-07-01

**Authors:** Gemma Parra, Ebbing Lautenbach, Mosepele Mosepele, Naledi Mannathoko, Robert Gross, Douglas R. Call, Brooke M. Ramay, Sylvia Omulo, C. P. Girish Kumar, Tarun Bhatnagar, Fahmida Chowdhury, Syeda Mah-E-Muneer, Rafael Araos, Jose M. Munita, Johanna Acevedo, Garrett Mahon, Rachel M. Smith, Ashley Styczynski

**Affiliations:** 1 Division of Healthcare Quality Promotion, Centers for Disease Control and Prevention, 1600 Clifton Road, MS A-24, Atlanta, GA 30329 USA; 2 University of Pennsylvania, Philadelphia, PA USA; 3 University of Botswana, Gaborone, Botswana; 4 Washington State University, Pullman, WA USA; 5 National Institute of Epidemiology, Chennai, India; 6 icddr,b, Dhaka, Bangladesh; 7 Facultad de Medicina Clinica Alemana, Universidad del Desarrollo, Santiago, Chile

**Keywords:** Antimicrobial resistance, Colonization, Surveillance, Epidemiologic trends, COVID-19, Bacterial infection, Epidemiology

## Abstract

The recognized burden of antimicrobial resistance (AR) is greatest in low- and middle-income countries (LMICs), but limitations in surveillance preclude accurate estimates of AR. We aimed to evaluate colonization in communities and hospitals across six LMICs for two clinically-important pathogens: extended-spectrum cephalosporin-resistant Enterobacterales (ESCrE) and carbapenem-resistant Enterobacterales (CRE). Participants in hospitals and communities provided rectal swabs or stool samples for ESCrE and CRE identification. Isolates recovered from selective agars underwent confirmatory identification and antibiotic susceptibility testing (AST) using Vitek^®^ 2, MALDI-TOF, and/or disc diffusion testing. ESCrE and CRE were defined based on established breakpoints of phenotypic resistance to third-generation cephalosporins and carbapenems, respectively, to calculate prevalence of colonization. Community prevalence estimates were weighted to account for sampling design differences. A total of 10,139 participants across the 6 countries provided samples; 63% were females with a median age of 35 years (range: 0–99). Colonization with ESCrE in hospitals was high in all sites (range 34–84%). In communities, ESCrE colonization ranged from 22 to 77%. Prevalence of CRE colonization in hospitals ranged from 7 to 36% and in communities ranged from < 1 to 14%. These findings reveal a high burden of AR colonization in LMICs in both communities and hospitals. Cost-effective strategies to reduce AR colonization burden are needed in LMICs.

**Supplementary Information:**

The online version contains supplementary material available at 10.1038/s41598-025-94750-3.

## Introduction

Antimicrobial resistance (AR) poses an escalating global health threat as a leading cause of infectious-disease-related deaths^1^. The United States Centers for Disease Control and Prevention (CDC) and the World Health Organization have classified several antimicrobial-resistant organisms as priority threats, including extended-spectrum beta-lactamase (ESBL)-producing Enterobacterales and carbapenem-resistant Enterobacterales (CRE)^2,3^. This classification is based on the increasing prevalence, association with adverse health outcomes, and limited antibiotic treatment options for infections caused by these pathogens^2,4^.

Extended-spectrum cephalosporin-resistant Enterobacterales (ESCrE) are mainly comprised of organisms that produce ESBLs, which mediate resistance to most beta-lactam antibiotics^5^. Infections caused by ESBL-producing Enterobacterales are associated with higher mortality and length of stay in hospitals and intensive care units (ICUs) compared to infections caused by non-ESBL-producing strains^6^. Although ESBL-producing organisms were first identified in healthcare settings, the incidence of community-onset infections caused by these bacteria is increasing^7^. Carbapenems are used as a last resort to treat infections caused by resistant Gram-negative bacteria (GNB) like ESCrE; however, strains of GNB that produce carbapenemases and are resistant to these antibiotics have emerged (i.e., CRE)^8^. Due to limited treatment options, infections caused by CRE can have a mortality rate of up to 50%^9^. Prior to 2000, CRE were relatively uncommon but have doubled in prevalence in the last decade among healthcare-associated infections and have a high risk for spread in the community due to the mobile nature of AR genes through plasmids^8^. Both ESCrE and CRE have traditionally been detected in healthcare settings but are now being increasingly detected in the community^5,8^. Although data from high-income countries have demonstrated an increase in infections with these pathogens both in and out of healthcare settings, evidence from low- and middle-income countries (LMICs) is sparse^7^.

Statistical modeling reveals that the burden of AR is greatest in LMICs, but the accurate characterization of AR is challenged by surveillance limitations^1^. Existing AR surveillance systems that depend on clinical isolates can lead to incomplete and biased estimates because they primarily capture data from severe cases not responding to empiric treatment in healthcare settings, neglecting milder infections and those occurring in community settings, thus skewing the true prevalence and distribution of resistance patterns^10^. The impact of this constraint is particularly pronounced in LMICs because of limitations in access to healthcare and diagnostic stewardship^11,12^. Moreover, the limited existing studies describing the burden of ESCrE and CRE in LMICs are primarily focused on healthcare settings and do not provide insights into community prevalence^11,12^.

One approach to overcome these limitations in clinically derived burden estimates is to evaluate colonization with AR, which can help mitigate the impact of barriers to healthcare access and limitations in diagnostic practices^10^. Additionally, colonization often precedes infection and can increase the risk of infection with the colonized antimicrobial-resistant pathogen in healthcare settings^13,14^ and communities^15^, making it an important stage in pathogenesis^10,13–16^. Moreover, undetected colonization can result in onward transmission of AR, leading to further dissemination in the population. Therefore, colonization is a valuable metric that can be used to understand the potential burden of disease and facilitate the prevention and control efforts in communities and healthcare settings. These data, especially from LMICs, are critical for understanding epidemiologic trends as well as for directing prevention efforts for these hard-to-treat antimicrobial-resistant pathogens.

Colonization burden of these clinically important resistant GNB also appears to be increasing. According to a global systematic review of colonization studies, there has been a 10-fold increase in colonization with ESBL-producing *E.coli* in the community and a 3-fold increase in healthcare settings over the past 20 years^17^. Generally, colonization with antimicrobial-resistant pathogens in healthcare settings is higher, but this review observed a faster-growing colonization rate in the community, with a 1.5% yearly increase^17^. Colonization prevalence was highest in Asia and Africa, while the Americas and Europe reported a lower but still significant prevalence^5,17^. The rising pressure of AR colonization in the community stresses the importance of understanding the epidemiology dynamics of these pathogens. The Antibiotic Resistance in Communities and Hospitals (ARCH) research consortium is the first multi-country study evaluating the population-based prevalence of ESCrE and CRE colonization among community-dwelling and hospitalized individuals^18^.

The goal of these analyses was to evaluate colonization prevalence of ESCrE and CRE across the six ARCH sites, which are located in low- and middle-income countries across three continents, intending to provide visibility into the burden of AR in settings underrepresented by AR surveillance.

## Methods

### ARCH study consortium

Between 2018 and 2022, researchers in six low- and middle-income countries (Bangladesh, Botswana, Chile, Guatemala, Kenya, and India) conducted a cross-sectional, population-based study involving the collection of specimens to assess colonization with high-threat antimicrobial-resistant pathogens. Botswana, Guatemala, and Kenya included both urban and rural locations and enrolled children as well as adults (except for the Botswana hospital setting). Botswana additionally enrolled patients from an outpatient clinic. Bangladesh, Chile, and India enrolled only adults from either urban or rural settings. For these analyses, we evaluated the prevalence of colonization with ESCrE and CRE. Individuals residing in a household within the sampling area were considered eligible for the study if they slept overnight in their household for at least four weeks and did not have a fever, diarrhea, or cough at the time of specimen collection. Hospitalized patients were eligible if they did not have severe neutropenia, diarrhea, or active gastrointestinal bleeding at the time of enrollment. Participants and/or their guardians provided written informed consent, and participants provided biological samples, either stool or rectal swabs. Isolates were recovered using selective media and underwent confirmatory identification and antibiotic susceptibility testing (AST) using Vitek^®^ 2, MALDI-ToF, and/or disc diffusion testing. The general ARCH protocol has been published, and any variations specific to each site can be found in supplementary Tables S1 and S2^19^. All participants who provided biological samples were included in the subsequent analysis.

### Statistical methods

To determine the prevalence of colonization, we classified ESCrE and CRE based on the Clinical and Laboratory Standards Institute (CLSI) AST standards for third-generation cephalosporins and carbapenems (31st edition, M100). ESCrE colonization was defined as isolation of Enterobacterales resistant to ceftazidime, ceftriaxone, and/or cefotaxime and susceptible to carbapenems in a stool specimen collected from a study participant^20^. CRE colonization was defined as isolation of Enterobacterales with resistance to at least one carbapenem (i.e., ertapenem, meropenem, doripenem, and/or imipenem) in a stool specimen collected from a study participant^20^. Participants could be considered positive for both ESCrE and CRE.

For each ARCH site, the prevalence of ESCrE or CRE was calculated by dividing the number of individuals who were determined to be colonized by the total number of participants sampled and tested. Community participants were sampled differently across sites; therefore, we adjusted for the varying sampling methods by calculating weighted prevalence estimates (Table S1). This allowed for a more robust comparison between prevalence estimates than those calculated by the individual study sites. None of the site estimates were intended to be representative of the entire country’s population but rather of the hospitals and the communities they serve. Three separate analyses were conducted, stratifying by study setting (hospital/community), age category (adults/children), and community participant type (urban/rural). Kenya, Botswana, and Guatemala were the only three sites that enrolled both adults and children and enrolled participants from both urban and rural communities. Participants in Botswana that were enrolled from an outpatient clinic were grouped with the community participants, given similarities in colonization prevalence. All tests performed were two-sided, and a *P*-value of < 0.05 was considered statistically significant. Additionally, 95% confidence intervals were calculated to assess the precision of the estimates and to interpret statistically significant differences between groups. All statistical analyses were performed using SAS version 9.4 (SAS Institute; Cary, NC).

## Results

Across the six participating countries, a total of 10,136 participants provided samples, with a median age of 35 years (interquartile range: 22–52) (Table 1). Of those, 6,247 (62%) of participants were female. The prevalence of colonization with ESCrE was higher among hospitalized participants than among community participants (Figure S1). Colonization among hospitalized participants was found to be greater than 80% in Bangladesh and India (Table 2). The lowest prevalence was observed in Chile and Botswana with approximately one-third of hospitalized participants colonized with ESCrE. Similar trends were observed in the community, with ESCrE colonization prevalence ranging from 22 to 76%.

**Table 1:** Cohort characteristics by ARCH country, 2018–2022.

Characteristics	Bangladesh	Botswana	Chile	Guatemala	India	Kenya	Total
Age (years) median (range)	35 (18–99)	32 (0–92)	58(16–100)	25 (0–93)	45 (18–85)	28 (0–91)	35 (0-100)
Sex n (%)							
Male	646 (45)	686 (28)	626 (55)	479 (39)	573 (44)	879 (34)	3889 (38)
Female	787 (55)	1783 (72)	506 (45)	743 (61)	740 (56)	1688 (66)	6247 (62)
Hospital unit* n (%)							
ICU	4 (1)	0 (0)	66 (9)	93 (15)	52 (9)	0 (0)	
Non-ICU	715 (99)	100 (100)	709 (91)	548 (85)	504 (91)	100 (100)	
Location** n (%)							
Urban		700 (35)		204 (35)		925 (54)	
Rural		1300 (65)		377 (65)		790 (46)	
Age groups ** n (%)							
Adults (≥ 18 years old)		1432 (72)		453 (78)		1308 (76)	
Children (< 18 years old)		568 (28)		128 (22)		407 (23)	
Sampling periods	Apr – Oct 2019	Jan – Sep 2020 except Apr – May	Dec 2018 – May 2019	Community: Nov 2019 – Mar 2020; Jul – Oct 2021 Hospital: Mar – Sep 2021	Nov 2020 – Mar 2022	Jan 2019- Mar 2020	
Hospital participant enrollment	1 tertiary care hospital	3 acute care hospitals	4 public hospitals in 4 cities	1 tertiary care hospital	2 tertiary government hospitals	4 hospitals	
Community participant enrollment	Capital city	Capital city and 2 semi-rural villages and 6 outpatient health clinics	1 semi-rural city	Multiple villages	1 semi-urban city	2 cities	

*Among hospitalized patients.

**Urban/rural data only available for community participants in Botswana, Guatemala, and Kenya. Age groups include both hospital and community participants < 18 except for the Botswana hospital setting.

**Table 2:** Prevalence of ESCrE and CRE colonization in hospital and community participants from 2018 through 2022 across ARCH sites.

ESCrE colonization prevalence						
ARCH site	Hospital			Community		
	N	%	95% CI	N	%	95% CI
Bangladesh	719	82.2	(79.4–85.0)	714	76.5	(72.7–80.2)
Botswana	469	35.6	(31.3–40.0)	2000	24.4	(22.5–26.3)
Chile	775	33.5	(30.2–36.9)	357	22.1	(17.8–26.5)
Guatemala	641	59.4	(55.6–63.2)	581	41.9	(36.8–47.0)
India	556	83.6	(80.4–86.6)	757	75.8	(72.6–79.0)
Kenya	852	63.8	(60.6–67.1)	1657	47.4	(44.7–50.1)

CRE colonization prevalence						
ARCH site	Hospital			Community		
	N	%	95% CI	N	%	95% CI
Bangladesh	719	35.5	(32.0–39.0)	714	8.9	(6.6–11.3)
Botswana	469	7.0	(4.7–9.4)	2000	0.6	(0.3–0.9)
Chile	775	13.5	(11.1–16.0)	357	4.5	(2.3–6.6)
Guatemala	641	33.2	(29.6–36.9)	581	1.5	(0.3–2.8)
India	556	22.7	(19.2–26.2)	757	14.2	(11.6–16.8)
Kenya	852	8.1	(6.3–9.9)	1657	1	(0.6–1.5)

CRE colonization was less prevalent than ESCrE colonization across all ARCH sites in both hospitals and communities (Table 2). CRE colonization was higher among hospitalized participants than community participants (Figure S2). One-third of hospitalized participants in India and Bangladesh were found to be colonized with CRE, whereas Botswana and Kenya had fewer than 10% of hospitalized participants colonized with CRE. The highest community prevalence of CRE colonization was in India (14%). Botswana, Kenya, and Guatemala each reported < 2% community CRE colonization.

In countries where adults and children were enrolled, ESCrE prevalence estimates were similar for both (Table 3). Among the three sites that collected data from urban and rural populations, ESCrE colonization was higher among urban participants. However, the difference was only statistically significant for Botswana and Kenya (P-value 0.003 and 0.03, respectively) (Table 3). The prevalence of CRE colonization was too low to make any comparisons by age groups or urban/rural status in these sites. The predominant organisms isolated across all sites were *Escherichia coli* and *Klebsiella pneumoniae* for both ESCrE and CRE isolates (Figures S3 and S4). E. coli accounted for 63–93% of ESCrE isolates across all ARCH sites in both community and hospital settings. K. pneumoniae contributed between 4 and 31% of ESCrE isolates. Among CRE isolates, E. coli represented 48–80%, while K. pneumoniae ranged from 0 to 42%. All other organisms are listed in the supplemental materials.

**Table 3:** Prevalence of ESCrE colonization by age category (adults/children) and community participant type (urban/rural) in Botswana, Guatemala, and Kenya (with Kenya only enrolling children < 5 years of age).

	Botswana*			Guatemala			Kenya		
	N (%)	95% CI	*p*-value	N (%)	95% CI	*p*-value	N (%)	CI	*p*-value
Adults	1432 (24.7)	22.4, 26.9	0.68	741 (51.8)	48.2, 55.4	0.28	1305 (47.4)	44.4, 50.4	0.98
Children	568 (28.8)	20.3, 27.3		481 (48.6)	44.2, 53.1		352 (47.3)	41.8, 52.9	

	Botswana			Guatemala			Kenya		
	N (%)	95% CI	*p*-value	N (%)	95% CI	*p*-value	N (%)	95% CI	*p*-value
Urban	700 (28.3)	24.9, 31.6	**0.003**	204 (45.6)	37.2, 53.9	0.24	884 (50.1)	46.3, 53.8	**0.03**
Rural	1300 (22.3)	20.0, 24.6		377 (39.3)	33.0, 45.6		773 (44.3)	41.5, 48.4	

*Botswana did not enroll children from their hospital population.

Statistically significant *p*-values are in bold.

## Discussion

In this multi-site study, we found a high prevalence of ESCrE colonization in hospitals across all sites. A global systematic review reported a colonization prevalence of 21% with ESBL-producing *E.coli* among inpatients in healthcare facilities^17^. In our study, the prevalence of ESCrE in hospitalized patients ranged from 33 to 84%. This difference is likely due to majority of the studies in the review being conducted in high-income countries. In line with findings from previous research, our study observed a higher prevalence of ESCrE colonization among hospital participants compared to community participants. This may be attributed to factors such as poor infection prevention and control (IPC) practices, overcrowding in healthcare facilities, which can facilitate transmission, lack of water and sanitation healthcare infrastructure, and antibiotic use^21^. Although we observed this relationship across our studies, sites like India and Bangladesh demonstrated nearly identical ESCrE colonization prevalence between the two settings (Table 2). This highlights the need for understanding drivers of antibiotic resistance in the community that may not be associated with healthcare settings.

ESCrE colonization was prevalent in communities across all sites. Like previous global AR colonization studies, we observed the highest community ESCrE colonization prevalence in South Asia. However, published estimates ranged from 22 to 46%, while we observed rates of 77% and 76% in Bangladesh and India, respectively^5,17^. This could be because both reviews included studies conducted between 1978 and 2018, during which there has been a documented increase in the prevalence of infection with ESBL-producing bacteria.

In all the ARCH hospital settings, we observed a significantly lower prevalence of CRE colonization compared to colonization with ESCrE. The prevalence of CRE colonization in hospitals was as high as 35%, which should raise concern given that prior to 2001, nearly all clinical Enterobacterales were susceptible to carbapenems^22^. CRE have even been detected in hospital settings in LMICs where carbapenems are not routinely used due to their high costs, highlighting the need for improved surveillance and epidemiological research^23^. In 2019, a global study conducted in 64 medical centers found a CRE resistance prevalence of 4.5%, and a multi-center study of long-term acute care facilities in the United States showed that 25% of *K. pneumoniae* isolates were resistant to carbapenems^24,25^. Although these data are not representative of the ARCH sites, they demonstrate that in high-income countries there has been observed increases in colonization and infection due to CRE in the last decade.

Prior studies on CRE colonization have reported prevalence in community settings ranging from < 1 to 5%^26,27^. In our study, CRE colonization prevalence in the community ranged from < 1 to 14%. Even in the sites with the lowest prevalence of CRE colonization in hospital participants, CRE was still detected in the community. The highly transmissible nature of plasmid-borne carbapenemases could account for its rapid global spread in and outside of hospitals. As demonstrated by the high prevalence of ESCrE in the community, there is a high potential for CRE to spread into the community, causing community-associated infections^8^. In contrast, in the United States, community-based studies have not detected CRE colonization except following international travel^28–30^. Although the data from the United States on CRE colonization in the community is sparse, the CDC’s Emerging Infection Program tracks CRE infection, and of the 4,996 CRE infections identified between 2016 and 2020, 20% were community-associated, suggesting the need for community interventions to interrupt transmission^31^.

The ARCH studies had varying enrollment periods (Table S1); therefore, half of the sites completed enrollment before the emergence of severe acute respiratory syndrome coronavirus 2 (SARS-CoV-2), while others began enrolling participants during the COVID-19 pandemic. Varying enrollment periods were not originally identified to be a variable of concern, but the COVID-19 pandemic disrupted healthcare systems, affected infection prevention and control processes, altered antibiotic use patterns, changed social interactions, and prompted notable shifts in movement and travel behaviors worldwide. Several studies in high-income settings reported an increase in resistant infections during the COVID-19 pandemic^32–35^. Modeling studies suggested an increased risk for colonization with antimicrobial-resistant bacteria in hospitals during SARS-CoV-2 outbreaks^36^, and one retrospective study in Italy observed an increase in CRE colonization from 7 to 50% among ICU patients from 2019 to March–April 2020^33^. The India ARCH site began data collection during the COVID-19 pandemic, whereas Botswana and Guatemala collected data both before and during the pandemic (Figure S5). The ARCH site in Botswana observed a decrease in community colonization rates following a national lockdown^37^. However, community sampling in Guatemala observed no differences in ESCrE or CRE colonization before and during the pandemic despite relatively stringent restrictions (Table S4)^38^. Thus, the pandemic may have had variable impacts on colonization prevalence estimates.

The geographical differences in colonization prevalence emphasize the need to identify regional factors contributing to these variations. While antibiotic use is one of the drivers of AR, there exist other contributing factors in the community, including poor water and sanitation, which can facilitate the spread of antimicrobial-resistant pathogens. Global modeling data and genomic studies have linked poor sanitation and contaminated potable water to the spread of AR^39,40^. According to The World Bank, Botswana and Chile were the only sites with basic sanitation coverage greater than 75% in 2020, and they were the sites with the lowest community prevalence of ESCrE colonization (24% and 22%, respectively) (Table 1)^41^. Urbanization has been found to be associated with a lower prevalence of AR, contrary to our findings where all community participants in urban areas had a higher ESCrE colonization prevalence than those in rural areas (Table 3)^40^. However, the referenced study included data from both high-income countries and LMICs, hypothesizing that urbanization may be indirectly linked with AR risk rather than indicative of improved sanitation infrastructure in urbanized areas. Additionally, exposure to animals could potentially contribute to this dynamic and may modify the urban/rural divide. Risk factor analyses conducted by the ARCH sites in Kenya and Botswana found animal contact to be associated with ESCrE colonization^42,43^. Further exploration of these regional differences may provide additional insights into the unique factors contributing to the rapid expansion of AR in the Global South^44^. Identifying the drivers of AR in communities is necessary to guide public health initiatives to combat AR spread. Aside from investing in IPC programs and better water and sanitation infrastructure in healthcare settings, there is an urgent need to invest in research to identify drivers and solutions to reduce AR colonization burden in communities. Not only does the expanding reservoir of AR in the community pose a significant threat, but it can also jeopardize any efforts of AR prevention and control in healthcare settings through IPC and antibiotic stewardship^44^.

A limitation of our study is the difference in enrollment methods across sites. Some sites sampled across multiple cities and towns from different regions and others only sampled from one area. Although we tried to address this limitation by adjusting for sampling methods, the variability in enrollment across sites limits our ability to compare between them. Other possible limitations are the variations in specimen type and laboratory analysis for confirmatory identification and AST (Table S2). Due to differences in sensitivity between these methods, there may be implications for the comparability of the data. However, we considered these potential trade-offs to be the most appropriate choice for the study sites, considering resources and other logistical constraints.

The detection of ESCrE and CRE across all the ARCH communities underscores the need to understand the relationship between and among hospital and community isolates to identify possible pathways of transmission. Characterizing genotypic determinants of resistance can help inform our understanding of genomic epidemiology, such as the prevalence of ESBL- and carbapenemase-producing genes, the role of plasmids in disseminating antibiotic resistance genes, and the co-selection that may be occurring among closely linked antibiotic resistance genes. Additionally, it can help with cluster identification in communities, which can guide population-based interventions. Lastly, there is a need to conduct longitudinal surveillance to observe temporal trends of these organisms to answer questions such as whether CRE, like ESCrE, will similarly become established in community reservoirs and lead to an increasing number of community-acquired infections. In conclusion, the high ESCrE and CRE burden in hospitals and communities in LMICs, which is much higher than what has been reported by high-income countries, calls for urgent actions to combat AR globally. This requires the development, financing, and implementation of national plans to combat AR using a One Health approach and the need to research strategies to reduce antimicrobial-resistant pathogen burden that are cost-effective and can be adopted by LMICs.

## Electronic supplementary material

Below is the link to the electronic supplementary material.

Supplementary Material 1.

## Data Availability

The datasets generated and analyzed during the current study are available from the corresponding author on reasonable request.
