# Supplementary material for: Colonization with antibiotic resistant bacteria in communities and hospitals across six countries, including Bangladesh, Botswana, Chile, Guatemala, India, and Kenya
Source: Sci Rep. 2025 Jul 1;15:21275. doi: 10.1038/s41598-025-94750-3 (PMC12218395; doi:10.1038/s41598-025-94750-3)

Supplementary materials

Supplementary table S1. Published methods and result differences for each ARCH study site.

| **ARCH Site** | **Enrollment periods** | **Community Sampling Methods** | **Sampling Weights*** | **Reason for differences in published prevalences** | **Ethical review boards** |
| --- | --- | --- | --- | --- | --- |
| Bangladesh | April – October 2019 | Two-stage cluster sampling | Calculated by authors | Did not calculate weighted prevalence rates. | Icddr,b Research Review Committee and Ethical Review Committee (#PR-18060) |
| Botswana | January – September 2020; except April – May | Snowball sampling | Not used | Used different phenotypic definitions | IRB of the University of Pennsylvania and the Botswana Ministry of Health and Wellness |
| Chile | December 2018 – May 2019 | Random sampling | Not used | Did not limit positive isolates to Enterobacterales. | Pontificia Universidad Catolica de Chile Comite etico cientifico CEC-MEDUC (# 18110003) and Comite Etico de la Investigacion Facultad de Medicinia Clinica Alemana-Universidad del Desarrollo (#2019-003) |
| Guatemala | Community: November 2019 – March 2020; July – October 2021  Hospital: March – September 2021 | Three-stage cluster sampling | Calculated by study site [1] | Included non-ARCH samples in their analysis. | Universidad del Valle de Guatemala Research Ethics Committee (#202-10-2019) |
| India | November 2020- March 2022 | Two-stage cluster sampling | Calculated by authors | Did not calculate weighted prevalence rates. | National Institute of Epidemiology Institutional Human Ethics Committee (NIE/IHEC/201904-02) |
| Kenya | January 2019 – March 2020 | Two-stage cluster sampling | Calculated by study site [2] | Did not calculate weighted prevalence rates. | Kenyatta National Hospital-University of Nairobi Ethics &Research Committee (KNH-ERC/A/228 and KNH/ERC/R/85) |

*Formula used by authors to calculate weights; Weight = $({\frac{\frac{\# of selected clusters}{total \# of clusters}}{\frac{\# sampled}{total \# in population sampled}})}^{-1}$

[1] Ita T, Luvsansharav UO, SmithRM, et al. Prevalence of colonization with multidrug-resistant bacteria in communities and hospitals in Kenya. Scientific Reports 2022; 12. https://doi.org/10.1038/s41598-022-26842-

[2] Ramay BM, Castillo C, Grajeda L, et al. Colonization with Antibiotic-Resistant Bacteria in a Hospital and Associated Communities in Guatemala: An Antibiotic Resistance in Communities and Hospitals (ARCH) Study. Clinical Infectious Diseases 2023; 77(Supplement_1): S82-S8. https://doi.org/10.1093/cid/ciad222

Supplementary table S2. Laboratory methods and results differences for each ARCH study site.

| **ARCH Site** | **Community specimen type** | **Hospital specimen type** | **Organism ID method** | **Antibiotic Susceptibility Testing method** |
| --- | --- | --- | --- | --- |
| Bangladesh | Stool, self collected | Stool, self-collection | Vitek2 | Vitek2 |
| Botswana | Rectal swab, collected by trained staff | Rectal swab, collected by trained staff | VITEK MS matrix-assisted laser desorption/ionization-time of flight (MALDI-TOF) | Vitek2 |
| Chile | Stool, self collected | Rectal swab, collected by trained staff | Matrix-assisted laser desorption/ionization-time of flight (MALDI-TOF) | Disk diffusion |
| Guatemala | Stool, self collected | Stool, self-collection | Vitek2 | Vitek2 |
| India | Stool, self collected | Stool, self-collection | Vitek2 | Vitek2 |
| Kenya | Stool, self-collection | Rectal swab collected by trained staff or stool, self collection | Vitek2 | Vitek2 |

Supplementary table S3. Prevalence estimates of ESCrE and CRE across countries weighted by sampling methods and unweighted, ARCH sites, 2018-2022.

|  | Weighted | | | Unweighted | | |
| --- | --- | --- | --- | --- | --- | --- |
| ESCrE | N | % | 95% CI | N | **%** | 95% CI |
| Bangladesh | 714 | 76.5 | (72.7 - 80.2) | 714 | 77.6 | (74.5 - 80.7) |
| Botswana | - | - | - | 2000 | 24.4 | (22.5 - 26.3) |
| Chile | - | - | - | 357 | 22.1 | (17.8 - 26.5) |
| Guatemala | 581 | 41.9 | (36.8 - 47.0) | 581 | 40.8 | (36.8 - 44.8) |
| India | 757 | 75.8 | (72.6 - 79.0) | 757 | 74.5 | (71.4 - 77.6) |
| Kenya | 1657 | 47.4 | (44.7 - 50.1) | 1715 | 47.8 | (45.4 - 50.2) |
| CRE | N | % | 95% CI | N | **%** | 95% CI |
| Bangladesh | 714 | 8.9 | (6.6 - 11.3) | 714 | 9.4 | (7.2 - 11.5) |
| Botswana | - | - | - | 2000 | 0.6 | (0.3 - 0.9) |
| Chile | - | - | - | 357 | 4.5 | (2.3 - 6.6) |
| Guatemala | 581 | 1.5 | (0.3 - 2.8) | 581 | 1.2 | (0.3 - 2.1) |
| India | 757 | 14.2 | (11.6 - 16.8) | 757 | 15.1 | (12.5 - 17.6) |
| Kenya | 1657 | 1 | (0.6 - 1.5) | 1715 | 1.3 | (0.8 - 1.9) |

*Sampling weights for Botswana and Chile not included due to differing methodologies.

Supplementary table S4. Prevalence estimates of ESCrE and CRE in ARCH sites that collected data prior to and during COVID-19 pandemic, 2019-2021.

|  | Community | | | | Hospital | | | |
| --- | --- | --- | --- | --- | --- | --- | --- | --- |
|  | **Pre-COVID** | | **COVID** | | **Pre-COVID** | | **COVID** | |
| Guatemala | % | 95% CI | % | 95% CI | % | 95% CI | % | 95% CI |
| ESCrE | 37.0 | (31.4, 42.7) | 43.6 | (35.3, 51.9) | N/A | | 59.4 | (55.6, 63.2) |
| CRE | 0.9 | (0.0, 1.9) | 2.0 | (0.0, 4.3) | NA | | 33.2 | (29.6, 36.9) |
|  | **Pre-COVID** | | **COVID** | | **Pre-COVID** | | **COVID** | |
| Botswana | % | 95% CI | % | 95% CI | % | 95% CI | % | 95% CI |
| ESCrE | 31.8 | (29.2, 36.4) | 20.3 | (18.1, 22.4) | 52.8 | (38.9, 66.7) | 31.5 | (26.9, 36.1) |
| CRE | 1.4 | (0.5, 2.3) | 0.2 | (0, 0.5) | 18.9 | (8.0, 29.8) | 4.6 | (2.5, 6.6) |

*Pre-COVID-19 defined as before March 11, 2020.

Supplementary figure S1. Prevalence of ESCrE in Community and Hospital Participants, ARCH sites, 2018-2022.

Supplementary figure S2. Prevalence of CRE in Community and Hospital Particpants, ARCH sites, 2018-2022.

Supplementary figure S3. Distribution of organisms among ESCrE Isolates, ARCH sites, 2018-2022*.

*Organisms by setting unavailable for Chile ARCH site.

** Other organisms isolated by country below.

**Bangladesh**: Community: Cronobacter sakazakii group 1, Klebsiella aerogenes, Enterobacter dissolvens, Enterobacter cloacae, Enterobacter cloacae complex, Morganella morganii, Salmonella enterica, Serratia ficaria, Shigella sonnei. Hospital: Klebsiella aerogenes, Enterobacter asburiae, Enterobacter dissolvens, Enterobacter cloaecae, Enterobacter cloacae complex, Morganella morganii.

**Botswana**: Community: Citrobacter braakii, Citrobacter freundii, Citrobacter sedlakii, Enterobacter cloacae, Enterobacter cloacae complex, Escherichia fergusonii. Hospital: Citrobacter braakii, Citrobacter freundii, Enterobacter cloacae, Enterobacter cloacae complex, Klebsiella oxytoca, Proteus mirabilis.

**Guatemala**: Community: Enterobacter aerogenes, enterobacter cloacae complex, klebsiella oxytoca. Hospital: Citrobacter freundii, Enterobacter aerogenes, enterobacter asburiae, enterobacter cloacae complex, escherichia fergusonii, klebsiella oxytoca, salmonella group, serratia odorifera.

**India**: Community: Citrobacter amalonaticus, Citrobacter sedlakii, Enterobacter cloacae complex, Klebsiella oxytoca, Klebsiella aerogenes, Morganella morganii. Hospital: Citrobacter amalonaticus, Citrobacter farmeri, Citrobacter sedlakii, Enterobacter cloacae comples, Klebsiella aerogenes, Morganella morganii.

**Kenya**: Community: Citrobacter freundii, Citrobacter koseri, Citrobacter youngae, Cronobacter sakazakii, Enterobacter aerogenes, Enterobacter cloacae complex, Klebsiella oxytoca, Shigella sonnei. Hospital: Citrobacter amalonaticus, Citrobacter freundii, Enterobacter cloacae cloacae, Enterobacter cloacae complex, Klebsiella oxytoca, Kluyvera intermedia, serratia plymuthica.

Supplementary figure S4. Distribution of organisms among CRE Isolates, ARCH sites, 2018-2022*.

*Organisms by setting unavailable for Chile ARCH site.

** Other organisms isolated by country below.

**Bangladesh:** Community: Enterobacter asburiae, Enterobacter cloacae cloacae, Morganella morganii, Morgenalla sibonii, Proteus mirabilis, Providencia stuartii. Hospital**:** Citrobacter freundii, Enterobacter cloacae dissolvens, Enterobacter cloacae cloacae, Enterobacter cloacae complex, Escherichia fergusonii, Klebsiella ozaenae, Kluyvera ascorbata, Morganella morganii, Morganella sibonii, Proteus hauseri, Proteus mirabilis, Providencia rettgeri, Providencia stuartii, Serratia fonticola, Shigella sonnei.

**Botswana:** Community**:** Enterobacter cloacae, Citrobacter sedlakii, Citrobacter amalonaticus. Hosptial**:** Citrobacter amalonaticus, Enterobacter cloacae, Enterobacter cloacae complex, Klebsiella oxytoca.

**Guatemala:** Community: Enterobacter cloacae complex. Hospital: Citrobacter amalonaticus, citrobacter farmeri, citrobacter freundii, enterobacter aerogenes, enterobacter cloacae complex, klebsiella oxytoca, proteus mirabilis, serratia ficaria.

**India:** Community: Enterobacter cloacae complex, Morganella morganii morganii, Providencia stuartii, serratia ficaria, serratia fonticola. Hosptial: Citrobacter sedlakii, enterobacter cloacae complex, Morganella morganii morganii, serratia ficaria.

**Kenya**: Community: Enterobacter cloacae cloacae, Enterobacter cloacae complex. Hospital: Enterobacter cloacae complex, Klebsiella oxytoca.

Supplementary figure S5. Enrollment timeline for ARCH sites.


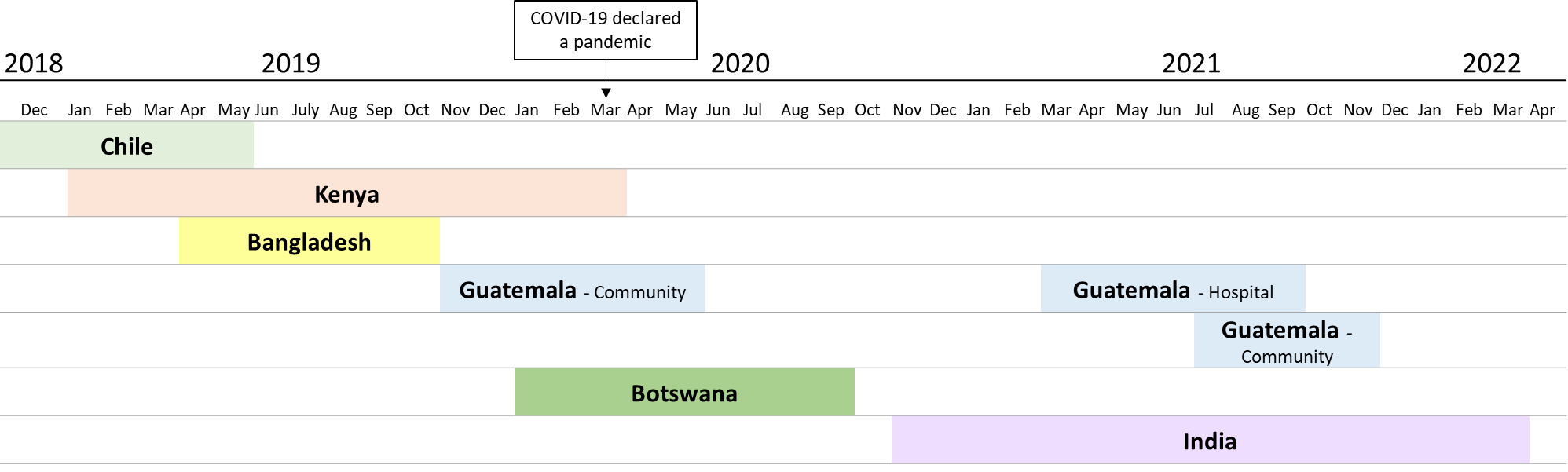

Supplement: Supplementary file 1 — Supplementary Material 1. [file 41598_2025_94750_MOESM1_ESM.docx]
